# CRISPR/Cas-Based Ex Vivo Gene Therapy and Lysosomal Storage Disorders: A Perspective Beyond Cas9

**DOI:** 10.3390/cells14151147

**Published:** 2025-07-25

**Authors:** Andrés Felipe Leal, Luis Eduardo Prieto, Harry Pachajoa

**Affiliations:** 1 Centro de Investigaciones en Anomalías Congénitas y Enfermedades Raras, Universidad Icesi, Cali 760031, Colombia; 2 Centro de Investigaciones Clínicas, Fundación Valle de Lili, Cali 760001, Colombia; luis.prieto.va@fvl.org.co; 3 Institute for the Study of Inborn Errors of Metabolism, Faculty of Science, Pontificia Universidad Javeriana, Bogotá 110231, Colombia; 4 Departamento de Genética Clínica, Fundación Valle de Lili, Cali 760001, Colombia

**Keywords:** CRISPR/Cas, ex vivo, gene therapy, lysosomal storage disorders

## Abstract

Lysosomal storage disorders (LSDs) are inherited metabolic conditions characterized by lysosomal enzyme deficiencies leading to substrate accumulation. As genetic diseases, LSDs can be treated with gene therapies (GT), including the CRISPR/Cas systems. The CRISPR/Cas systems enable precise and programmable genome editing, leading to targeted modifications at specific genomic loci. While the classical CRISPR/Cas9 system has been extensively used to generate LSD disease models and correct disease-associated genetic alterations through homologous recombination (HR), recently described Cas proteins as well as CRISPR/Cas9-derived strategies such as base editing, prime editing, and homology-independent targeted integration (HITI) offer a novel way to develop innovative treatments for LSDs. The direct administration of the CRISPR/Cas9 system remains the primary strategy evaluated in several LSDs; nevertheless, the ex vivo CRISPR/Cas9-based approach has been recently explored, primarily in central nervous system-affecting LSDs. Ex vivo approaches involve genetically modifying, in theory, any patient cells in the laboratory and reintroducing them into the patient to provide a therapeutic effect. This manuscript reviews the molecular aspects of the CRISPR/Cas technology and its implementation in ex vivo strategies for LSDs while discussing novel approaches beyond the classical CRISPR/Cas9 system.

## 1. Introduction

Lysosomal storage disorders (LSDs) are metabolic conditions characterized by overaccumulation of partially or non-degraded substrates due to impaired lysosomal function [1]. Primary LSD care involves enzyme replacement therapy (ERT) and symptom treatment to improve the patient’s quality of life [2]. Strategies such as substrate reduction, degradation therapy, and molecular chaperones could become potential treatments in the future [3,4]. As genetic diseases, LSDs can be treated via gene therapy (GT) to correct the mutation causing the disease. Although classical GT enables the delivery of therapeutic genes via adeno-associated virus (AAV) and lentivirus (LV), the vector dilution effect and random integration are significant challenges associated with these strategies [5,6,7].

**Figure 1:**
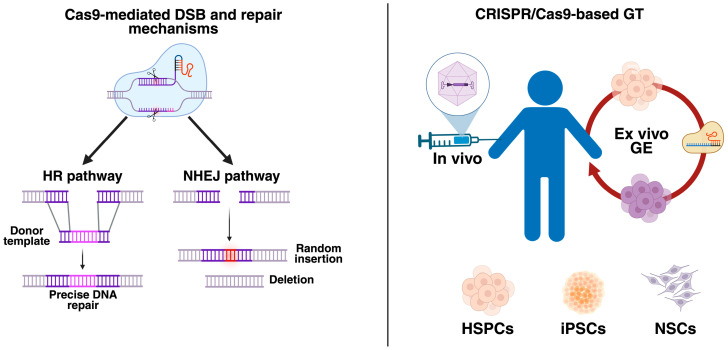
The classical CRISPR/Cas9 system and its use as a gene therapy (GT) approach. (**Left panel**) The classical CRISPR/Cas9 system requires a double-strand break (DSB) within the DNA mediated by an RNA-guided Cas9 protein. Upon DSB, the cell activates two central repair mechanisms: the homologous recombination (HR) and non-homologous end-joining (NHEJ) pathways. While HR is predominant in dividing cells, the NHEJ is active in both dividing and non-dividing cells. Most importantly, HR needs a donor template with homologous arms to be inserted into the host genome. Without a donor template, the NHEJ mediates the DSB repair, producing random insertions or deletions known as indels. (**Right panel**) The CRISPR/Cas9 system can be used for in vivo and ex vivo genome editing (GE). Likewise, current studies have demonstrated the suitability of hematopoietic stem progenitor cells (HSPCs), induced pluripotent stem cells (iPSCs), and neural stem cells (NSCs) for ex vivo CRISPR/Cas9-based GTs. This figure was created with Biorender.com.

Contrary to the AAV- and LV-based GTs, the clustered regularly interspaced short palindromic repeats (CRISPR) and CRISPR-associated proteins (Cas), abbreviated as CRISPR/Cas, lead to modifying the genome through precise DNA cutting and then harnessing natural homologous recombination (HR) DNA repair in the presence of a donor template (Figure 1). The absence of a donor template results in indel (insertion or deletion) formation due to the predominant activity of the non-homologous end-joining (NHEJ) pathway in mammalian cells [8,9]. While in vivo CRISPR/Cas9-based GT has demonstrated promising outcomes in several LSD models [10,11,12], the pre-existing anti-Cas9 immune response could limit its therapeutic efficacy [13,14]. In consequence, ex vivo CRISPR/Cas9-based GT has emerged as a novel alternative for several LSDs [15].

Most of the ex vivo CRISPR/Cas9 approaches tested for LSDs rely on modifying hematopoietic stem progenitor cells (HSPCs) in a controlled laboratory environment and later transplanting them into LSD animal models [15]. Although not all LSDs affect the central nervous system (CNS), about two-thirds of patients present neurological compromise [16]. In this regard, HSPCs transplantation (HSCT) has shown encouraging outcomes in CNS-affecting LSDs, such as mucopolysaccharidosis (MPS) I and metachromatic leukodystrophy, as it can induce medullary repopulation [17]. Relevantly, HSPCs can differentiate into all hematological precursors, including macrophages, which can cross the blood–brain barrier (BBB) and transform into microglia in response to the brain’s microenvironment [18]. A significant increase in BBB permeability appears to favor the migration of macrophages from the bloodstream into the CNS [19,20]. Likewise, it has been described that HSCT can be successfully implemented in some non-CNS-affecting LSDs, such as MPS IVA, as it improves the 6-minute walk test, increases stature, and enhances respiratory function and hearing in early-treated MPS IVA patients [21,22]. In this case, mature lysosomal enzyme-producing blood cells can distribute throughout the body and mediate cross-correction, thereby ameliorating systemic symptoms in tissues distant from the bone marrow (BM), such as the spleen, liver, and heart, by decreasing substrate accumulation, which ultimately increases patient survival [23].

HSCT is a promising alternative for treating LSDs; however, graft-versus-host disease (GVHD) is a significant concern that limits allogeneic HSCT even in the presence of matched human leukocyte antigen (HLA) [24]. Interestingly, using the CRISPR/Cas system, it is possible to isolate patients’ HSPCs, carefully engineer them in the laboratory, and finally reinfuse them into the patient to repopulate the BM (Figure 1) [15]. This autologous HSCT procedure significantly reduces the risk of GVHD and graft rejection [24]. While HSPCs are the most common cells used in ex vivo CRISPR/Cas-based GTs, the feasibility of induced pluripotent stem cells (iPSCs) and neural stem cells (NSCs) has also been demonstrated for some LSDs (Figure 1). Although the CRISPR/Cas9 system is the most common strategy for ex vivo approaches in LSDs, successful gene editing requires the generation of double-strand breaks (DSBs) within the DNA by Cas9 [25], which raises critical safety concerns, including potential unwanted Cas9 cutting. Thus, alternatives such as CRISPR/Cas12a, base editing (BE), prime editing (PE), and homology-independent targeted integration (HITI) could offer innovative options, improving the safety and efficiency of the CRISPR/Cas-based genome editing (GE).

This review summarizes the classical ex vivo CRISPR/Cas9 approaches for LSDs while exploring novel alternatives beyond Cas9 and HR for inducing genomic DNA rewriting. A comprehensive literature search was performed using PubMed, Web of Science, and Google Scholar databases. The search included peer-reviewed articles published between January 2015 and May 2025, with a focus on the most recent developments (2020–2025), using the Boolean operators (“CRISPR/Cas” OR “CRISPR-based editing”) AND (“ex vivo gene therapy” OR “hematopoietic stem cells” OR “induced pluripotent stem cells”) AND (“lysosomal storage disorders” OR “MPS” OR “Gaucher” OR “Fabry” OR “Krabbe”) AND (“base editing” OR “prime editing” OR “Cas12” OR “Cas13” OR “HITI”).

## 2. The CRISPR/Cas Systems

**Figure 2:**
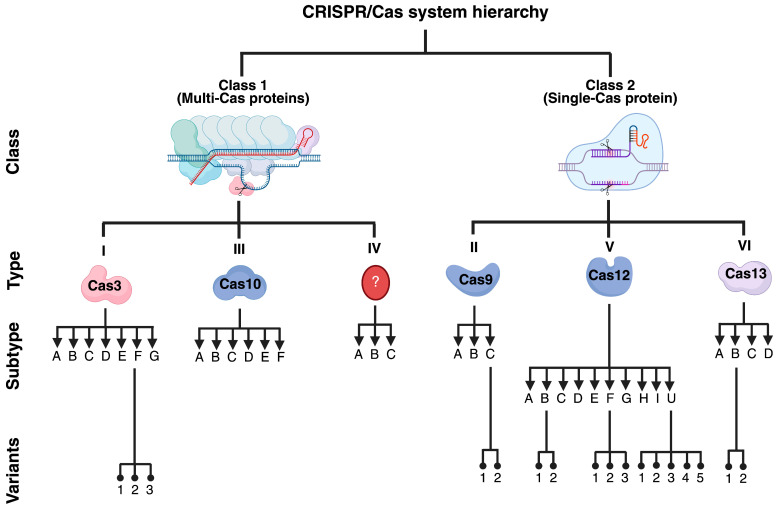
Classification of the CRISPR/Cas systems. The CRISPR/Cas system is primarily classified based on the presence of multi- or single-Cas proteins. Due to its simplicity, Cas proteins belonging to class 2 are the most explored for GE purposes; nevertheless, some class I proteins (i.e., Cas3) are also potential tools for treating human diseases. This figure was created with Biorender.com.

The CRISPR/Cas system is an adaptive immune system in prokaryotes [8,9]. The locus CRISPR harbors short repetitive elements separated by spacers and a series of CRISPR-associated (Cas) genes that encode for Cas proteins. During an invasion by bacteriophages, transposons, or plasmids, the CRISPR/Cas locus is activated to induce Cas-mediated cutting of foreign DNA [8]. The released DNA fragments are later inserted into the CRISPR locus as new spacers, conferring variability to the system. Upon re-infection by the same invader, a CRISPR-associated RNA (crRNA) recognizes the foreign genetic material and mediates Cas-dependent cleavage, thereby protecting the host [26]. The CRISPR/Cas systems are classified into two classes, six types, thirty-three subtypes, and seventeen variants (Figure 2) [27].

### 2.1. DNA-Targeting Cas Enzymes

While Cas9 remains the most popular DNA-binding endonuclease used in CRISPR/Cas, several other Cas proteins have been described and could be considered for GE purposes. The main features of Cas proteins are detailed in Table 1.

#### 2.1.1. Cas9

The Cas9 enzyme is the most commonly used endonuclease in CRISPR/Cas. The Cas9 enzyme is composed of a recognition and a nuclease lobe. Two catalytic domains, termed HNH (histidine–asparagine–histidine) and RuvC, mediate the DSB of dsDNA upon protospacer-adjacent motif (PAM) recognition [8,9,28]. Several modifications of the Cas9 protein have been reported, including high-fidelity Cas9 (HF-Cas9), Cas9 nickase (nCas9, lacking endonuclease activity from HNH or RuvC), and dead Cas9 (dCas9) as well as NHEJ-inhibiting proteins such as Brex27, CtIP, DN1S, and RAD52, among others, which are fused to Cas9 for enhancing the HR pathway [8,9]. We have published a comprehensive review of Cas9 variants and encourage readers to consult it [8].

#### 2.1.2. Cas3

The Cas3 enzyme is an effector enzyme with both endonuclease and helicase activity, predominantly binding ssDNA in a 3′-to-5′ direction upon exerting its helicase activity [29,30]. Contrary to Cas9, which induces small deletions [31], Cas3 “shreds” DNA, producing large deletions up to multiple kilobases from the starting point. The Cas3-mediated DNA processing results in fewer off-target effects compared to those produced by Cas9 [32,33]. Cas3 has been previously tested by Morisaka et al. (2019) in an exon-skipping strategy using Duchenne muscular dystrophy (DMD)-induced pluripotent stem cells (iPSCs) [34]. DMD is a genetic disorder caused by pathogenic variant mutations in the dystrophin gene, resulting in a deficiency of the dystrophin protein, which is essential for maintaining muscle function [35]. Results from this study demonstrated the recovery of the dystrophin protein through CRISPR/Cas3-mediated skipping of exon 45 [34], providing proof of concept for the therapeutic applicability of the CRISPR/Cas3 system.

#### 2.1.3. Cas12

Cas12a, initially classified as Cpf1, is a DNA endonuclease that generates staggered breaks resulting in 5 bp overhangs [36]. This is particularly relevant for HR, as it generates ssDNA, leading to access to the HR machinery. Most interestingly, Cas12a is approximately one-third the size of Cas9, making it more suitable for therapeutic applications where package size is limited, such as AAV vectors [37]. Although Cas12a has been used for the diagnosis of infection by SARS-CoV-2 [38,39], it has also been tested for GE applications. For instance, Cas12a has demonstrated a therapeutic impact in restoring the expression of the *CFTR* gene in an in vitro model of cystic fibrosis, resulting in an efficiency of up to 8%. Still, Cas12 resulted in lower efficacy compared to Cas9 [40]. Although Cas12e [41,42,43] and Cas12j [43,44] have been reported, Cas12f1 has garnered greater attention due to its smaller size compared to its counterparts [45,46]. Cas12f1 was recently tested by Cui et al. (2024) [47] to knock out the Nr2e3 gene in a mouse model of retinitis pigmentosa using AAV vectors. Upon a single subretinal injection in two-week-old mice, the authors found over 70% transduction efficiency in retinal cells and about 41% indel frequency [47]. Notably, mice showed improved cone function at one month post-treatment and an increased outer nuclear layer [47], suggesting that retinal degeneration was rescued upon CRISPR/Cas12f1 treatment.

#### 2.1.4. Cas14

Cas14 is the smallest Cas protein that can only bind and cleave ssDNA in a PAM-independent way [48]. Unlike Cas12a, Cas14 recognizes ssDNA with high specificity, a feature that has been a key factor in detecting single-nucleotide polymorphisms and differentiating between pathogens [49]. Contrary to other Cas proteins, Cas14 is susceptible to mismatches in the middle of the target region (~6 bp) [50], making it unsuitable for GT purposes.

### 2.2. RNA-Targeting Cas Enzymes

Most approaches observed in CRISPR/Cas utilize Cas proteins that can bind and cut DNA. Nevertheless, RNA-targeting Cas enzymes can also be implemented in clinical practice. Since these Cas enzymes do not target DNA but RNA, they can be used for transient knockdown.

#### 2.2.1. Cas7-11

Cas7-11 is a single effector that cuts ssRNA with minimal cell toxicity and lower off-target effects in mammalian cells compared to its counterpart, Cas13 [51,52]. Cas7-11 comprises a putative Cas11 domain and several Cas7 subunits [52,53]. As a recently discovered Cas enzyme, only a few pieces of evidence are available showing its potential in a transient knockdown. Moreno-Sánchez et al. (2025) [54] reported using CRISPR/Cas7-11 to suppress mRNA from the developmental genes *nanog* and *no-tail* in zebrafish. Interestingly, the CRISPR/Cas7-11 system demonstrated an in vivo efficiency of 60.1% without collateral activity [54]. Most importantly, when targeting the green fluorescent protein (GFP), the authors observed no developmental defects, 28S rRNA cleavage, or transcriptomic alterations in zebrafish embryos [54], supporting the safety of the CRISPR/Cas7-11 system.

#### 2.2.2. Cas13

Similar to Cas7-11, Cas13 is also an ssRNA-cutting endonuclease. Unlike Cas7-11, Cas13 exhibits collateral activity that mediates unintended, non-targeted RNA cutting [55,56]. The CRISPR/Cas13 system was recently tested by Kumar et al. (2024) [57] as a GT for retinal neovascularization by targeting VEGF mRNA in cell-derived retinal organoids and humanized VEGF transgenic mice. In the mouse model of proliferative retinopathy, the intravitreal delivery of the CRISPR/Cas13 system packaged into an AAV2 vector resulted in a ~50% reduction in human VEGF expression, along with a reduced leakage area, lower leaky vessel density, and fewer branch points in treated mice, suggesting an amelioration of disease progression [57]. No decrease in mouse VEGF was observed, supporting the high specificity of the system. Despite these promising achievements, the CRISPR/Cas13 system induced dysregulation of 23 unidentified genes, as detected by transcriptomic analysis [57], consistent with the reported collateral effect of Cas13 [55]. Novel variants of Cas13, such as CRISPR-DjCas13d, are under development to maintain the high specificity of Cas13 while eliminating its collateral effects [54].

### 2.3. CRISPR/Cas-Derived Alternatives

**Figure 3:**
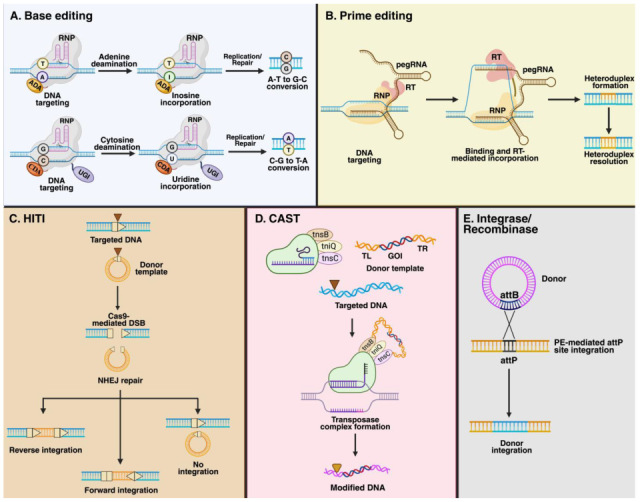
CRISPR/Cas9-based approaches. (**A**) BE is a genome editing tool that involves a ribonucleoprotein (RNP) complex, comprising an sgRNA and nCas9 protein. nCas9 is fused to either adenine (ADA) or cytosine (CDA) deaminase. Upon Cas9-mediated ssDNA nicking, ADA or CDA mediates the deamination of adenine and cytosine. While adenine deamination leads to inosine formation (upper), cytosine deamination leads to uracil (bottom). Later replication or reparation mechanisms allow the final conversion from I to G and U to T. (**B**) The PE system retains the RNP complex comprising a pegRNA and nCas9. nCas9 is fused to a reverse transcriptase (RT). The pegRNA is a large sgRNA containing a primer binding site (PBS) and an RT template. While the PBS leads to targeted sequence hybridization, the RT uses the RT template to synthesize the new gene information. The cell’s repair mechanism later corrects the mismatch between edited and non-edited DNA strands. (**C**) HITI is intended to integrate foreign DNA within host DNA using the NHEJ pathway. Both the target DNA and the donor template should contain sgRNA and PAM sequences to undergo Cas9-mediated cutting. Upon Cas9-mediated DSB generation, the NHEJ leads to donor insertion in forward or reverse directions. If forward direction occurs, the sgRNA and PAM sequences are abolished, impeding Cas9 cutting. Conversely, if a donor template is inserted in the reverse direction, the presence of the sgRNA and PAM sequences will lead to Cas9 cutting. Repair without donor template insertion will induce additional Cas9-mediated cleavage. (**D**) The CRISPR-associated transposases (CAST) system enables the insertion of a gene of interest (GOI) through the activity of Tn7-like transposons. CAST requires the presence of an RNP comprised of Cas12k and a sgRNA to recognize and bind the targeted DNA, along with a donor template flanked by transposon left (TL) and right (TR) sequences. Cas12 brings the Tn7-like genes to the target site. Later, DNA recognition by the RNP complex leads to the formation of a transposase complex, which ultimately enables the excision and insertion of the donor template, approximately 60 bp from the PAM sequence (GTN), within the target DNA [9,59]. (**E**) Gene insertion can also be achieved through the use of integrases/recombinases, which utilize the specific recombination sites attB and attP. In this approach, the presence of the attP sequence in the target DNA is achieved through the use of PE. Consequently, the presence of attB sites in the donor template later leads to the recombination of the GOI within the targeted DNA, resulting in a knock-in without inducing DSBs [9,59]. This figure was created with Biorender.com.

Initially, the CRISPR/Cas system was designed to modify the genome via Cas9-mediated DSB and later HR activation. Emerging approaches aim to edit the genome by leveraging enzymes such as deaminases and reverse transcriptase in CRISPR/Cas-based systems, which are referred to as base and prime editing, respectively (Figure 3). Similarly, the HR dependence for GE can be dismissed when working with the homology-independent targeted integration (HITI) system (Figure 3), potentially increasing the efficacy of the CRISPR/Cas systems in both dividing and non-dividing cells [58]. Finally, new DNA repair-independent targeted integration alternatives such as CRISPR-associated transposases (CAST) and those combining PE and integrases/recombinases have extended the CRISPR/Cas-based tools that can be evaluated as GT approaches (Figure 3).

#### 2.3.1. Base Editing

The base editors (BEs) are an alternative to the CRISPR/Cas system, based on the fusion of a Cas9 enzyme carrying mutations in its catalytic domains, RuvC and HNH [60]. Those mutations maintain PAM recognition and DNA targeting while abolishing Cas9-mediated DSB within the DNA. nCas9 is also suitable for base editing. Fused to Cas9, a base-modifying enzyme such as cytosine or adenine deaminase enables the system to convert cytosine to uracil and adenine to hypoxanthine (inosine), respectively [60,61]. While uracil is converted into thymine, hypoxanthine is converted into guanine during DNA replication and repair. Primary described BEs can only catalyze nucleotide transitions, purine–purine (A–G), and pyrimidine–pyrimidine (C–T), which limits their use for correcting transversions; nevertheless, new advances make correcting transversions via glycosylase base editors and adenine transversion editors possible [60]. Recently, a new generation of BEs has been developed, including Cas12a, which can mediate base editing in mammalian cells [62]. Cas12a can process multiple sgRNAs from a single array transcript [63], thus allowing multi-gene targeting. This could be an interesting alternative when several mutations affect a gene or different genes are involved.

#### 2.3.2. Prime Editing (PE)

PE is a CRISPR/Cas alternative that leads to insertions, deletions, and substitutions without introducing DSBs in the DNA. PE relies on the nCas9-mediated nicking and activity of the reverse transcriptase (RT) [64]. Contrary to the classical CRISPR/Cas9 system, the sgRNA is a prime editing guide RNA (pegRNA). While the sgRNA is a single, short RNA sequence, a pegRNA is a longer RNA containing a reverse-transcription template and a primer binding site at the 3′ end [65]. The RT uses the template placed on the pegRNA to synthesize new DNA, which will later be introduced into the cell’s DNA. Since its first description, PE has undergone extensive modifications aimed at improving RT efficiency (PE2, PE6, and PEmax), resolving mismatches (P3, P4, and P5), refining sizing, optimizing codons (PEmax), and increasing pegRNA stability (PE7) [65,66,67,68]. Like BEs, PE has also incorporated Cas12, enabling the system to target multiple genes in human cells [69].

#### 2.3.3. Homology-Independent Targeted Integration (HITI)

HITI is a CRISPR/Cas9-based system that enables the knock-in of foreign DNA without relying on HR, unlike the classical CRISPR/Cas9 system [70]. In HITI, the donor template contains the desired insertion sequence, flanked by the sgRNA and PAM sequences, which are recognized by the Cas9 protein. Consequently, Cas9 will recognize and cut both the targeted DNA and the donor template, enabling the NHEJ repair mechanism [58]. Upon NHEJ repair, forward-inserted sequences will abolish the sgRNA/PAM sequence, resulting in a permanent insertion [58,70]. In contrast, reverse orientation restores the previous Cas9-mediated sgRNA/PAM cut sequences, allowing Cas9 to recut until a forward orientation is achieved.

## 3. CRISPR/Cas9: Ex Vivo Approaches in LSDs

The CRISPR/Cas9 system has been successfully evaluated in some LSDs using ex vivo approaches, including MPS I, MPS IVA, Gaucher, Krabbe, and Fabby. Table 2 summarizes the main findings for these ex vivo CRISPR/Cas9-based strategies.

### 3.1. Mucopolysaccharidosis (MPS) I

MPS I (OMIM 607014) is a multi-systemic LSD caused by an alpha-L-iduronidase (IDUA; EC 3.2.1.76) deficiency that leads to lysosomal accumulation of dermatan sulfate (DS) and heparan sulfate (HS) [79,80]. MPS I is a central nervous system (CNS)-affecting LSD, also producing skeletal dysplasia along with cardiac and ocular manifestations [80]. Initial proof-of-concept studies using CRISPR/Cas9 and iPSCs were conducted by Miki et al. (2019) [71]. In this study, mouse embryonic fibroblast-derived iPSCs were established from an MPS I mouse model and edited with the CRISPR/Cas9 system to remove a *Neo^R^* gene inserted into exon VI of the *Idua* gene. The authors successfully observed the recovery of IDUA expression in CRISPR/Cas9-edited iPSCs, with no difference from WT iPSCs [71]. Although no significant changes were detected in *Oct4*, *Nanog*, and *Sox2* genes in teratoma formation assays using CRISPR/Cas9-edited iPSCs compared to WT iPSCs [71], the authors discussed the potential tumorigenicity of the iPSCs and CRISPR/Cas9 off-targeting as critical concerns before implementing this approach in clinical practice.

In 2019, Goméz-Ospina et al. demonstrated the suitability of an ex vivo CRISPR/Cas9-based GT in an MPS I mouse model. By electroporating human CD34+ cells with a CRISPR/Cas9 system targeting the human CCR5 locus, the authors successfully achieved supraphysiological IDUA expression levels in human CD34+ cells without compromising their stemness and in vivo engraftment properties [72]. Upon transplantation of edited CD34+ cells into immunodeficient MPS I mice, a significant increase in the IDUA activity in serum, liver, spleen, and brain was observed. A consistent GAG reduction and recovery of bone thickness of the zygomatic, skull, and femur were observed. Likewise, CRISPR/Cas9-modified CD34+ cells decreased the isolectin B4 and glial fibrillary acidic protein (GFAP) staining in mouse brains, suggesting that gliosis is ameliorated [72]. The molecular validation of this CRISPR/Cas9 system revealed an indel occurrence rate of less than 0.5% across 62 predicted off-target sites, supporting the safety of the sgRNA used in human CD34+ cells.

Sub-lethal irradiation and busulfan-mediated myeloablation are standard protocols for conditioning before HSCT [15,20,81], and their effectiveness in enhancing the engraftment of CRISPR/Cas9-modified human CD34+ cells was assessed by Poletto et al. (2022) in MPS I mice. Interestingly, the authors reported that busulfan-mediated myeloablation significantly improved the engraftment of CRISPR/Cas9-modified human CD34+ cells in BM, long-term homing in BM-derived visceral cells [20], and the greater migration of BM-derived cells in the brain compared to the irradiation protocol. Consistently, an improvement in IDUA expression and GAG clearance was observed in busulfan-treated mice compared to irradiation [20], highlighting the importance of preconditioning regimens before transplantation of CRISPR/Cas9-modified human CD34+ cells.

### 3.2. Mucopolysaccharidosis (MPS) IVA

MPS IVA (OMIM 253000) is a systemic LSD caused by a deficiency of the N-acetylgalactosamine-6-sulfatase (GALNS; EC 3.1.6.4) enzyme. GALNS leads to the lysosomal degradation of keratan sulfate (KS) and chondroitin 6-sulfate (C6S) [80,82]. MPS IVA patients are characterized by skeletal dysplasia, cardiopulmonary, and ocular complications [83]. Recently, Herreño-Pachón et al. (2025) reported, for the first time, the use of a novel CRISPR/nCas9-based GT to modify human CD34+ cells [73]. In this study, the authors designed paired sgRNAs targeting the human AAVS1 locus to knock in an expression cassette carrying the GALNS cDNA [73]. Upon electroporation of the CRISPR/nCas9 system into human CD34+ cells, the authors found that these cells retained their stemness, including the ability to proliferate and differentiate [73]. Moreover, upon co-culture of CRISPR/nCas9-modified CD34+ cells with human MPS IVA fibroblasts, a significant increase in GALNS activity was observed in MPS IVA fibroblasts, suggesting that human CD34+ cells-derived GALNS enzyme was successfully uptaken by human MPS IVA fibroblasts. Most importantly, the authors reported a significant decrease in lysosomal mass and mono-KS, supporting the intracellular sorting of GALNS into the lysosome [73]. The authors also found a recovery in global and mitochondrial-dependent oxidative stress. This study also revealed a significant increase in mitochondrial mass and a pro-apoptotic profile in untreated MPS IVA fibroblasts, which was efficiently restored to WT levels upon co-culture with CRISPR/nCas9-modified CD34+ cells [73], paving the way for a novel ex vivo CRISPR/nCas9-based GT.

### 3.3. Gaucher

Gaucher disease (GD) is an LSD that belongs to the sphingolipidoses and results from the deficiency of β-glucocerebrosidase (GCase; EC 3.2.1.45). GCase degrades glucosylceramide (GlcCer) and glucosylsphingosine (GlcSph) within the lysosome [84,85]. GD subtypes include nonneuronopathic (GD1; OMIM 230800), acute neuronopathic (GD2; OMIM 230900), and chronic neuronopathic (GD3; OMIM 231000). GlcCer and GlcSph accumulate primarily within mononuclear phagocyte cells, which are identified in the BM, spleen, and liver as wrinkled, paper-like cells termed Gaucher cells [85]. An ex vivo CRISPR/Cas9-based strategy was tested by Scharenberg et al. (2020) as a potential alternative for treating GD [74]. By targeting human CCR5, the authors successfully integrated an expression cassette carrying GCase cDNA, driven by the lineage-specific CD68 promoter, to restrict its expression to the monocyte/macrophage lineage [74]. The use of the spleen focus-forming virus (SFFV) promoter to drive GCase expression in human CD34+ cells induced toxic effects, underscoring the importance of careful promoter selection [74]. Upon CRISPR/Cas9 GE, the human CD34+ cells could differentiate into CD14+/Cd11b+ cells, suggesting successful human monocyte/macrophage differentiation. CRISPR/Cas9-edited CD34+ cells did not fail to differentiate into all hematopoietic lineages [74], supporting the maintenance of stemness upon knock-in. Most importantly, transplantation into immunodeficient mice demonstrated higher engraftment and self-renewal capacity upon serial transplantation [74], further supporting the preservation of stemness.

Given that GlcCer accumulation primarily occurs in the monocyte/macrophage lineage, it is not surprising that monocyte/macrophage functions, such as migration, phagocytosis, and activation, are impaired [86,87]. A recent study conducted by Ramalingam et al. (2023) [75] investigated the feasibility of using the CRISPR/Cas9 system to correct the L444P (1448T→C) mutation in GD2 iPSCs by targeting this mutation at the *GBA* gene. iPSCs-derived macrophages resulted in GCase-expressing cells and the restoration of motility and phagocytosis of zymozan [75]. It has been observed that GD protects against *Mycobacterium tuberculosis* infection [88]. Interestingly, the recovery of GCase expression increased the susceptibility of iPSC-derived macrophages to *M. tuberculosis* infection [75], revealing new considerations when attempting CRISPR/Cas9-mediated GE for GD.

### 3.4. Krabbe

Krabbe disease (OMIM 245200) is an LSD caused by a deficiency of galactocerebrosidase (GALC; 3.2.1.46). This deficiency leads to the lysosomal accumulation of galactosylceramide (GalCer) in Schwann and oligodendroglial cells, thereby affecting the central and peripheral nervous systems [89]. Although CD34+ cells and iPSCs are the most common sources tested for ex vivo CRISPR/Cas9-based GT, a study by Dever et al. (2019) [76] showed the feasibility of neural stem cells (NSCs) as a potential alternative for treating Krabbe disease. In this study, three loci, IL2RG, HBB, and CCR5, were targeted via CRISPR/Cas9 to insert an expression cassette carrying a functional copy of GALC in NSCs. The highest HR was achieved when targeting IL2RG (~6%), while the lowest HR was observed with CCR5 [76]. Upon subventricularly transplanting CRISPR/Cas9-edited NSCs into immunodeficient neonatal mice, the authors noticed engraftment in the cortex and corpus callosum, along with migration from the subventricular zone to the olfactory bulb and the rostral migratory stream [76], suggesting that CRISPR/Cas9-edited NSCs preserve their biological properties in vivo. Significantly, the co-culture of Krabbe fibroblasts and GALC-expressing NSCs resulted in Krabbe fibroblasts’ mannose 6-phosphate-dependent GALC uptake [76]. Although functional assessment of phenotype recovery in Krabbe fibroblasts was not performed upon co-culture, the intracellular GALC increase is expected to exert a therapeutic effect. Upcoming experiments in Krabbe animal models, such as the Twitcher mouse model, are still to be conducted to validate this novel ex vivo CRISPR/Cas9-based GT preclinically.

### 3.5. Fabry

Fabry disease (FD; OMIM 301500), an LSD caused by α-galactosidase A (GLA; EC 3.2.1.22) deficiency, is characterized by the accumulation of globotriaosylceramide (Gb3) and Lyso-GB3 in the kidneys, heart, brain, and peripheral nervous system [90]. A recent study published by Choi et al. (2023) [77] used the CRISPR/Cas9 system to correct the mutation 1268fs*1 (c.803_806del) in FD iPSCs. While GLA enzyme activity was significantly recovered upon GE, CRISPR/Cas9-edited iPSCs retained pluripotency-associated marker expression, a normal karyotype, and displayed differentiation into three germ layers, with no detectable off-target effects [77], thereby developing an ex vivo CRISPR/Cas9 proof-of-concept to correct the deletion of the *GLA* gene. Similar outcomes were recently reported by Karl-Schöller et al. (2025) in FD iPSCs for the mutation c.1069C>T in the *GLA* gene [78]. While the CRISPR/Cas9 system led to the restoration of the WT sequence, the authors also observed the clearance of Gb3 deposits, along with the preservation of pluripotency markers, further supporting the therapeutic effect of the CRISPR/Cas9 for FD. The transplantation of these CRISPR/Cas9-edited iPSCs in animal models of Fabry disease still needs to be carried out to test their therapeutic efficacy in vivo.

## 4. Beyond Cas9 Enzymes and HR: An Overview in Non-LSD Models

Most ex vivo CRISPR/Cas-based GT approaches in LSDs utilize Cas9 and HR as the primary molecular mechanisms to knock in expression cassettes at safe harbors, thereby expressing the missing lysosomal enzyme and mediating cross-correction. Nevertheless, some non-LSD studies have demonstrated the feasibility of Cas proteins beyond Cas9, achieving transgene expression and/or do not depend on DBS/HR. This section describes recent non-LSD studies evaluating non-Cas9 proteins and non-classical HR-based knock-in approaches in ex vivo CRISPR/Cas-based alternatives. Table 3 compiles ex vivo CRISPR/Cas approaches in non-LSD models.

### 4.1. CAR-T Cells: Implementing Cas12a

Chimeric antigen receptor (CAR)-T cell therapy is an innovative ex vivo treatment option for cancer, particularly hematological malignancies [97]. Patients’ T cells are isolated via leukapheresis and engineered with a CAR construct to express synthetic receptors on the surface of the T cell. These receptors will recognize and suppress specific malignancy-antigen-expressing tumor cells [98,99]. Even though the Food and Drug Administration (FDA) has recently approved several CAR-T-cell alternatives for B-cell lymphoma, B-cell acute lymphoblastic leukemia (B-ALL), mantle cell lymphoma, and follicular lymphoma [100], cytokine release syndrome (CRS) and immune effector-cell associated neurotoxicity syndrome (ICANS) are significant concerns that still need to be addressed [101]. To overcome this, some studies have utilized the CRISPR/Cas system to edit CAR-T cells, aiming to enhance their function, develop universal CAR-T cells, and improve their safety.

Early studies by Ling et al. (2021) [91] demonstrated the efficiency of the CRISPR/Cas12a system in enhancing the knock-in of an anti-CD19-containing expression cassette at the endogenous T-cell receptor (TCR) locus in primary T cells. While wtCas12 showed about 30% knock-in efficiency, a Cas12 covalently conjugated to the 5′ terminus of crRNA (cCas12) enhanced the knock-in efficiency up to 52.7% [91], suggesting that this chemical modification increases the Cas12 stability. Major concerns in CAR-T cell preparations arise from patients’ poor quality and quantity of T cells [102]; thus, creating universal CAR-T cells is now a promising research field. As Cas12 enables multi-gene targeting, the study conducted by Ling et al. (2021) [91] also evaluated the simultaneous CRISPR/Cas12-mediated knock-out of TCR, β2 microglobulin (β2M), PD1, and CTLA4, along with the anti-CD19-containing expression cassette knock-in. Interestingly, cCas12 showed up to 7.4-fold GE four-gene knock-out efficacy improvement compared to wtCas12, while cCas12 led to 37% GE anti-CD19-containing expression cassette knock-in, which was higher than that observed with wtCas12 (28%) [91]. These interesting results show the potential of ex vivo CRISPR/Cas12-mediated multi-gene targeting.

The AAVS1 locus is a well-known safe harbor for inserting large expression cassettes [103]. We have extensively tested the AAVS1 locus in several LSDs using human fibroblasts [104,105,106], and we recently evaluated its feasibility for inserting a GALNS-containing expression cassette in CD34+ cells [73]. In a study by Mohr et al. (2023) [92], the AAVS1 locus was used for inserting a GFP-containing expression cassette under the control of the promoters JET (195 bp), PGK (511 bp), EF-1α (1195 bp), and CAG (1723 bp), which differ in size, using the CRISPR/MAD7 system in Jurkat cells. MAD7 is a modified Cas12 released by Inscripta in 2017 that requires a crRNA and identifies AT-rich genome regions, resulting in staggered DNA cutting [107]. In this study, the authors reported up to 30% CRISPR/MAD7-mediated GE HR efficiency, regardless of the promoter evaluated [92], suggesting that insert size did not influence knock-in efficiency. Later experiments assessed the insertion of a CAR expression cassette at the AAVS1 locus in primary T cells using CRISPR/MAD7-mediated GE [92]. Although a high GE efficiency of up to 85% was observed in primary T cells at 13 days after GE, it was dramatically reduced (~30%) upon CRISPR/MAD7-mediated multi-gene knock-out targeting of CTLA4, PDCD1, TIGIT, CD247, PTPN6, or PTPN11 [92], which are common targets for enhancing and invigorating CAR-T cells [108].

### 4.2. Hematological Disorders: Base and Prime Editing

Ex vivo BE and PE have been tested in several hematological disorders to modify hematopoietic stem progenitor cells (HSPCs), which can differentiate into all hematological progenitors and virtually reach any tissue in the body. The first FDA-approved CRISPR/Cas9-based GT (Casgevy) was developed for sickle cell disease (SCD) and was approved in December 2023 [109]. Casgevy is a GT based on disrupting the *BCL11A* gene via Cas9-mediated DSB, which ultimately favors the expression of γ-globin to compensate for the absence of functional β-globin affected by mutations on the *HBB* gene [110].

An ABE was evaluated by Newby et al. (2021) [93] to correct a point mutation in the β-globin gene (*HBB*) in HSPCs from patients with SCD. The ex vivo delivery of an mRNA ABE resulted in the 80% conversion of the pathogenic *HBB^S^* (CAC) into a non-pathogenic *HBB^G^* (CGC) known as Makassar. HSCT in immunodeficient mice achieved up to 68% *HBB^G^* as well as a 5-fold decrease in BM reticulocytes following hypoxia-induced sickling [93]. Moreover, Makassar globin represented up to 79% of β-globin after 16 weeks post-transplantation into humanized mice. Transplanted mice also exhibited normalization of hematological parameters and a reduction in splenomegaly compared to untreated mice [93]. Contrary to what was observed with some ex vivo CRISPR/Cas9 GTs [111], ABE did not activate p53 or large deletions in BE HSPCs [93]. Similar findings have been achieved in β-thalassemia patient-derived HSPCs [112], with a new generation of ABEs termed ABE8e [113]. Importantly, using mRNA to deliver ABEs leads to higher off-target effects than the ribonucleoprotein complex delivery [112], which is commonly associated with the persistence of the BE within the cell.

Although studies such as those conducted by Newby et al. (2021) reported using BE to modify *HBB^S^* to *HBB^G^* [93], the transversion causing *HBB^S^* (A>T) cannot be directly reverted through BE [60]. Instead, PE is suitable for modifying this type of mutation [66], overcoming the limitations of BE. In 2023, Everette et al. [94] showed the correction of *HBB^S^* (A>T) to WT *HBB^A^* with a frequency of up to 41% in HSPCs from patients with SCD when using PE3max. Interestingly, *HBB^A^* was found in ~42% of erythroblasts and reticulocytes at 17 weeks post-transplantation, while HSPC-derived erythrocytes resulted in 28–43% normal hemoglobin [94]. Although PE leads to the recovery of the transversion causing *HBB^S^* to WT *HBB^A^*, the GE efficiency observed by Everette et al. (2023) [94] was significantly lower (43%) than that reported by Newby et al. (2021) [93] for BE (79%) when modifying *HBB^S^* by *HBB^G^*. Regardless of the efficacy, both BE and PE offer novel alternatives to modify any mutation with lower risks than the classical CRISPR/Cas9 system.

### 4.3. HITI: Advancing in Proof-of-Concept Assays

Current ex vivo HITI-based GE using ex vivo assays has tested the efficacy of the HITI system for inserting genes at specific genomic regions. In 2021, Bloomer et al. [96] evaluated the insertion of GFP at the first non-coding exon of the *ITGB2* gene. Mutations within the *ITGB2* gene, which encodes for the β2 integrin subunit CD18, produce leukocyte adhesion deficiency type 1 (LAD-1), an inherited immunodeficiency. In this study, the authors found that while 63% of insertions occurred in a forward direction, approximately 11% of bulk CD34+ cells exhibited GFP positivity from day 14 to 28 post GE, suggesting stable gene integration [96]. No significant changes in differentiation potential from HITI-edited CD34+ were noticed when compared with untreated CD34+ cells. Most interestingly, the transplantation of HITI-edited CD34+ cells in immunodeficient mice resulted in up to 21% of GPF-positive CD45+ cells, supporting the engraftment of HITI-edited CD34+ cells [96]. The therapeutic effect of HITI-edited CD34+ cells on LAD-1 still needs to be addressed.

Similar outcomes were reported by Byambaa et al. (2021) when using the HITI system to insert an Il2rg exon 2–8 cDNA-containing cassette into the intron 1 of the *Il2rg* gene in mouse HSCs [95]. Disruption of the *Il2rg* gene produces X-linked severe combined immunodeficiency (X-SCID) [114]. Up to 18.7% of knock-in efficacy and stemness preservation were reported upon HITI treatment. When transplanting HITI-edited mouse HSCs in an X-SCID mouse model, 3 out of 13 recipients showed an increase in white blood counts and T- and B-cell development after 8 weeks post transplantation compared to untreated mice [95]. In those three mice, CD45+ CD3+ T cells resulted in the expression of IL2rg in peripheral blood, BM, and thymus, supporting the therapeutic effect of the ex vivo HITI-based CRISPR/Cas9 system [95].

## 5. Conclusions and Future Perspectives

Undoubtedly, the CRISPR/Cas systems have opened novel alternatives for treating genetic diseases, including LSDs. While it is clear that CRISPR/Cas9 is the most common approach used in ex vivo strategies, implementing non-Cas9 proteins could become an innovative alternative. The CAR-T technology has demonstrated that higher GE efficiencies can be achieved with Cas12a than with Cas9 at several safe harbors, including the AAVS1 locus. The AAVS1 locus has been evaluated in several LSDs, and recently, we tested it for editing CD34+ cells using a CRISPR/nCas9-based GT approach for treating MPS IVA [73]. Further experiments comparing the efficacy of the Cas12a, wtCas9, and nCas9 should be performed to establish the higher GE strategy. Most interestingly, Cas12a can mediate multi-gene targeting, offering a new avenue for attempting gene insertion at multiple loci simultaneously (i.e., CCR5, AAS1, Album intron 1, etc.), which could lead to higher lysosomal enzyme expression than that observed when targeting a single locus.

On the other hand, given that Cas9-mediated DSB can induce unwanted DNA cutting, implementing DSB-free strategies such as PE could offer a promising alternative to correct mutations in LSDs, as PE can correct both transitions and transversions. Indeed, an advanced PE variant, PE3max, has shown comparable outcomes for stemness preservation and genome editing efficiency to those observed with CRISPR/Cas9 in HSPCs [94], making it feasible to substitute the classical CRISPR/Cas9 system. Notably, it is essential to highlight that hundreds of mutations can affect genes involved in lysosomal function, limiting the universal use of a single PE-based GT. Nonetheless, some mutations are commonly expressed, thus enabling the use of PE to treat frequent mutations.

Finally, because primary CD34+ HSPCs can undergo quiescent phenotypes in which NHEJ is the primary DSB repair mechanism [96,115], the HITI system could offer a new avenue for editing HSPCs. This premise has been tested in some proof-of-concept experiments; therefore, upcoming preclinical studies are necessary to establish the real therapeutic effect of an ex vivo HITI-based GT for treating LSDs. Likewise, HITI relies on Cas9-mediated DSB, raising concerns about its potential off-target effect. Consequently, low off-target scoring sgRNA and high-fidelity Cas9 enzymes should always be attempted, along with rigorous next-generation sequencing, to validate the absence of unwanted Cas9 cutting while preserving the higher efficiency of HITI in quiescent primary cells.

## Figures and Tables

**Table 1:** Main features of Cas proteins.

* Cas Variant	MW (kDa)	Substrate Preference	PAM Requirement	Collateral Activity	Editing Mechanism
Cas9	160	dsDNA	5′-NGG-3′	No	Blunt-end DSBs
Cas3	120	ssDNA	Varies (Cascade-dependent)	No	Processive DNA degradation
Cas12a (Cpf1)	130	dsDNA	5′-TTTV-3′	Yes (ssDNA)	Staggered DSBs
Cas 12e (CasX)	112	dsDNA	5′-TTCN-3′	No	ssDNA cleavage
Cas12j (CasΦ)	95	ssDNA	PAM-independent	Yes (ssDNA)	Staggered DSBs
Cas12f1	60	ssDNA	T-rich	Yes (ssDNA)	ssDNA cleavage
Cas14	50	ssDNA	PAM-independent	Yes (ssDNA)	ssDNA cleavage
Cas7-11	120	ssRNA	PFS-like motifs	No	RNA cleavage
Cas13	150	ssRNA	PFS	Yes (ssRNA)	RNA cleavage

* Alternative names given to Cas variants are presented in parentheses. **MW**, molecular weight; **PAM**, protospacer-adjacent motif; **PFS**, protospacer-flanking site.

**Table 2:** Ex vivo CRISPR/Cas9-based approaches in LSD models.

Model	CRISPR/Cas System Tested	Cell Type	Locus	IE (%)	OT (%)	Eng. (%)	Stemness	Ref.
MPS I	CRISPR/Cas9	iPSCs	IDUA	2.7	NR	NA	Preserved	[71]
MPS I	CRISPR/Cas9	HSPCs	CCR5	54	<0.5	21.5	Preserved	[72]
MPS I	CRISPR/Cas9	HSPCs	CCR5	TBI:5.7 BU:20	NR	* TBI:45 * BU:73	NR	[20]
MPS IVA	CRISPR/nCas9	HSPCs	AAVS1	10	ND	NA	Preserved	[73]
Gaucher	CRISPR/Cas9	HSPC	CCR5	29.9	NR	23.2	Preserved	[74]
Gaucher	CRISPR/Cas9	iPSCs	GBA	NR	ND	NA	Preserved	[75]
Krabbe	CRISPR/Cas9	NSC	IL2RG	2.8	<0.7	NA	Preserved	[76]
Krabbe	CRISPR/Cas9	NSC	CCR5	2.4	NR	NA	NR	
Fabry	CRISPR/Cas9	iPSCs	GLA	NR	NR	NA	Preserved	[77]
Fabry	CRISPR/Cas9	iPSCs	GLA	NR	ND	NA	Preserved	[78]

BM, bone marrow; BU, busulfan; Eng., engraftment in BM (CD45+); HSPCs, hematopoietic stem progenitor cells; IE, insertion efficiency; iPSCs, induced pluripotent stem cells; NA, not applicable; ND, not detected; NR, not reported; NSC, neural stem cells; OT, off-target; TBI, total body irradiation. * Median (min, max). Note: While most of the cell models correspond to human-derived stem cells, results from the study [71] were derived from mouse-derived stem cells.

**Table 3:** Ex vivo CRISPR/Cas-based approaches in non-LSDs.

Model	CRISPR/Cas System Tested	Cell Type	Locus	IE (%)	OT (%)	Eng. (%)	Stemness	Ref.
CAR-T	CRISPR/wtCas12	T cells	TCR	30	NR	NA	NA	[91]
	CRISPR/cCas12			52.7				
CAR-T	CRISPR/MAD7	T cells	AAVS1	85	ND	NA	NA	[92]
			* AAVS1	30				
SCD	ABE	HSPCs	HBB	80	<2	70	Preserved	[93]
SCD	PE3max	HSPCs	HBB	41	<1	97	Preserved	[94]
X-SCID	HITI	HSPCs	IL2RG	18.7	ND	13.2	Preserved	[95]
LAD-1	HITI	HSPCs	ITGB2	12	ND	21	Preserved	[96]

ABE, adenine base editing; Eng., engraftment in BM (CD45+); HITI, homology-independent targeted integration; HSPCs; hematopoietic stem progenitor cells; IE, insertion efficiency; LAD-1, leukocyte adhesion deficiency type 1; NA, not applicable; ND, not detected; NR, not reported; OT, off-target; PE3max, prime editing; SCD, sickle cell disease; X-SCID, X-linked severe combined immunodeficiency. * AAVS1 + multi-gene targeting at AAVS1 and CTLA4, PDCD1, TIGIT, CD247, PTPN6, or PTPN11. Note: While most of the cell models correspond to human-derived stem cells, results from the study [95] were derived from mouse-derived stem cells.

## Data Availability

No new data were created or analyzed in this study.

## Abbreviations

The following abbreviations are used in this manuscript:

BE	Base editing
BM	Bone marrow
DSB	Double-strand break
GAG	Glycosaminoglycan
GE	Genome editing
GT	Gene therapy
HR	Homologous recombination
HSCT	Hematopoietic stem cell transplantation
HSPCs	Hematopoietic stem progenitor cells
iPSCs	Induced pluripotent stem cells
NHEJ	Non-homologous end joining
PE	Prime editing
WT	Wild type
